# Interventions to reduce low-value imaging – a systematic review of interventions and outcomes

**DOI:** 10.1186/s12913-021-07004-z

**Published:** 2021-09-18

**Authors:** Elin Kjelle, Eivind Richter Andersen, Lesley J. J. Soril, Leti van Bodegom-Vos, Bjørn Morten Hofmann

**Affiliations:** 1 Institute for the Health Sciences at the Norwegian University of Science and Technology (NTNU) at Gjøvik, NTNU Gjøvik, Postbox 191, 2802 Gjøvik, Norway; 2 Department of Community Health Sciences and The Health Technology Assessment Unit, O’Brien Institute for Public Health, University of Calgary, 3280 Hospital Dr NW, Calgary, Alberta T2N 4Z6 Canada; 3 Medical Decision making, Department of Biomedical Data Sciences, Leiden University Medical Center, P.O. Box 9600, 2300 RC, Leiden, the Netherlands; 4 Centre of Medical Ethics, University of Oslo, Postbox 1130, Blindern, 0318 Oslo, Norway

**Keywords:** Low-value, Diagnostic imaging, Radiology, Reduce, Inappropriate, Intervention

## Abstract

**Background:**

It is estimated that 20–50% of all radiological examinations are of low value. Many attempts have been made to reduce the use of low-value imaging. However, the comparative effectiveness of interventions to reduce low-value imaging is unclear. Thus, the objective of this systematic review was to provide an overview and evaluate the outcomes of interventions aimed at reducing low-value imaging.

**Methods:**

An electronic database search was completed in Medline – Ovid, Embase-Ovid, Scopus, and Cochrane Library for citations between 2010 and 2020. The search was built from medical subject headings for Diagnostic imaging/Radiology, Health service misuse or medical overuse, and Health planning. Keywords were used for the concept of reduction and avoidance. Reference lists of included articles were also hand-searched for relevant citations. Only articles written in English, German, Danish, Norwegian, Dutch, and Swedish were included. The Mixed Methods Appraisal Tool was used to appraise the quality of the included articles. A narrative synthesis of the final included articles was completed.

**Results:**

The search identified 15,659 records. After abstract and full-text screening, 95 studies of varying quality were included in the final analysis, containing 45 studies found through hand-searching techniques. Both controlled and uncontrolled before-and-after studies, time series, chart reviews, and cohort studies were included. Most interventions were aimed at referring physicians. Clinical practice guidelines (*n* = 28) and education (n = 28) were most commonly evaluated interventions, either alone or in combination with other components. Multi-component interventions were often more effective than single-component interventions showing a reduction in the use of low-value imaging in 94 and 74% of the studies, respectively. The most addressed types of imaging were musculoskeletal (*n* = 26), neurological (*n* = 23) and vascular (*n* = 16) imaging. Seventy-seven studies reported reduced low-value imaging, while 3 studies reported an increase.

**Conclusions:**

Multi-component interventions that include education were often more effective than single-component interventions. The contextual and cultural factors in the health care systems seem to be vital for successful reduction of low-value imaging. Further research should focus on assessing the impact of the context in interventions reducing low-value imaging and how interventions can be adapted to different contexts.

**Supplementary Information:**

The online version contains supplementary material available at 10.1186/s12913-021-07004-z.

## Background

The rapidly expanding use of health services is challenging and health care expenditures are mounting [1]. This has underscored the need for more efficient use of finite healthcare resources. However, according to the Organization for Economic Co-operation and Development (OECD), approximately 10–34% of health service spending is potentially inappropriate, representing ineffective and wasteful use of health care resources [2]. Such services are referred to as low-value care, which is defined “*an intervention in which evidence suggest it confers not or very little benefit for patients, or risk of harm exceeds probable benefit or, more broadly, the added costs of the intervention do not provide proportional added benefits*” [3].

While diagnostic imaging provides crucial information for the diagnostics of patients [4], inappropriate or low-value imaging are estimated to constitute 20–50% of radiological examinations internationally [2, 5–8]. Several interventions to reduce low-value imaging have been evaluated in the literature, including guidelines such as iRefer, iGuide, as well as national and international initiatives such as the National Institute for Health and Care Excellence (NICE) “Do-not-do list,” and the Choosing Wisely campaign [9–12]. However, the effect of such efforts on low-value diagnostic imaging has been limited due to barriers such as financial incentives, practice behavior, self-referral, lack of feedback, patient expectations, and duplicate imaging examinations [5, 11, 13–17]. Some interventions even seem to increase the use of inappropriate imaging [18, 19].

Several approaches to address the use of inappropriate health services, beyond low-value imaging, have been extensively evaluated. Education or training programs for health care personnel, clinical decision support, feedback, patient education, shared decision making, and economic incentives are but a few examples [5, 20–24]. However, the great quantity and variability of available approaches makes it unclear which measures are most suitable to target low-value imaging and overutilization. While research on interventions to reduce low-value care, in general, recommend implementation of multi-component interventions in complex health care systems [12, 25–28] there is still uncertainty as to why or when an intervention will be effective in diagnostic imaging specifically and/or in which clinical circumstances they are effective. Earlier systematic reviews on interventions in imaging have addressed specific interventions as image sharing or clinical decision support systems or specific imaging examinations or patient complaints [26, 29–32]. However, there is no encompassing systematic review assessing the outcome of various types of interventions to reduce low-value imaging. Thus, the objective of this systematic review was to provide an overview and evaluate the outcomes of interventions aimed at reducing low-value imaging.

## Methods

This systematic review was conducted based on the Preferred Reporting Items for Systematic Reviews and Meta-analyses (PRISMA) statement (PROSPERO ID: CRD42020208072). The electronic database search was developed in Medline – Ovid (Table 1) and further adapted to Embase-Ovid, Scopus, and Cochrane Library. The terms used were built from medical subject headings (MESH) for Diagnostic imaging/Radiology, Health service misuse/Medical overuse, and Health planning. Keywords were used for the concept of reduction/avoiding. Also, the search was broadened with text word and keyword synonyms. The complete search strategy is available in Additional file 1. Searches were carried out in September and October 2020; last search made on 13th October 2020. Papers written in English, German, Danish, Norwegian, Dutch, and Swedish were eligible and language filters were used to exclude other languages. Keywords were used to exclude studies on animals, mass screening, and unnecessary care besides imaging services. No other limitations were applied.

**Table 1:** Search strategy developed in Medline (Ovid)

#	Medline Ovid
1	diagnostic imaging/ or cardiac imaging techniques/ or imaging, three-dimensional/ or neuroimaging/ or radiography/ or radionuclide imaging/ or respiratory-gated imaging techniques/ or tomography/ or ultrasonography/ or whole body imaging/
2	exp Radiology/
3	(MRI or x-ray* or xray* or ultrasound* or mammography or ultrasonography or DEXA or DXA or CT or radiograph* or radiolog* or tomography or imaging).tw.
4	(CAT adj scan).tw.
5	(bone adj scan).tw.
6	(Magnetic adj resonance adj imaging).tw.
7	1 or 2 or 3 or 4 or 5 or 6
8	exp Health Services Misuse/ or exp. Medical Overuse/
9	(Unnecessar* or overuse* or Inappropriate* or wasted or low-value or overdiagn* or overutili* or misuse* or (Low adj value) or unwarrent or redundant).tw.
10	(Choosing adj wisely).tw.
11	8 or 9 or 10
12	7 and 11
13	Animal/ not (animal/ and human/)
14	12 not 13
15	limit 14 to ((danish or dutch or english or german or norwegian or swedish) and last 10 years)
16	exp Health Planning/
17	(reduc* or prevent* or stop* or replac* or abandon* or avoid* or deinvest* or de-invest or deadopt* or de-adopt* or deimplement* or de-implement* or restrict* or lower* or decrease* or (practice adj revers*) or educat* or guidel*).tw.
18	(academic adj detailing).tw.
19	16 or 17 or 18
20	15 and 19
21	exp Mass Screening/
22	(Unnecessary adj surger*).tw.
23	(unnecessary adj biops*).tw.
24	(mammography adj screening).tw.
25	(lung adj cancer adj screening).tw.
26	(unnecessary adj invasive adj procedure).tw.
27	(prenatal adj screening).tw.
28	(case adj report).tw.
29	(comment or editorial or letter).pt.
30	(Radioactive adj Waste).tw.
31	(machine adj learning).tw.
32	(deep adj learning).tw.
33	(radio adj therapy).tw.
34	(optical adj imaging).tw.
35	(soil or cell* or fetal or dentist* or denture*).tw.
36	(cancer adj screening).tw.
37	21 or 22 or 23 or 24 or 25 or 26 or 27 or 28 or 29 or 30 or 31 or 32 or 33 or 34 or 35 or 36
38	20 not 37

### Eligibility criteria

Primary empirical studies assessing interventions to reduce the use of low-value diagnostic imaging examinations were included. Studies designed as randomized controlled trials, non-randomized trials, descriptive studies, mixed-methods studies, and qualitative studies were included. While systematic reviews and meta-analyses were not included, the reference lists of relevant systematic reviews and meta-analyses were hand-searched for additional primary studies for inclusion. Studies published before 2010 were excluded due to the changes in perception on low-value imaging through the preparation and introduction of the Choosing Wisely campaign in 2012. The inclusion and exclusion criteria are provided in Table 2.

**Table 2:** Inclusion and exclusion criteria for assessing record eligibility

Inclusion criteria	Exclusion criteria
Empirical study	Published before 2010 or after 2020
Assessing interventions aimed to reduce the use of low-value diagnostic imaging	Dental imaging, optical imaging, thermal imaging, microscopic imaging
Outcome of interventions to reduce low-value diagnostic imaging	Patient case reports, letter, comment
English, German, Dutch, Danish, Swedish, and Norwegian language	Mass-screening related studies

### Selection of records and methodological quality appraisal

The records were archived using Thomson Reuters EndNote X9.3.3 library and duplicates were removed. All remaining records were transferred to Rayyan QCRI [33] where titles and abstract review (EK and BMH) and full-text review and quality assessment (EK, ERA, LJJS, LvB-V and BMH) were completed by two teams of reviewers. Each study was quality assessed by one reviewer and double checked by EK for consistency. The Mixed Methods Appraisal Tool (MMAT) was used to assess the methodical quality of all included studies as it is considered to be an appropriate tool for appraisal of interventional studies of different methodologies [34]. Any disagreements during abstract or full-text screening were resolved through discussion and consensus. Reference lists of included articles were also hand-searched for relevant articles for inclusion. A grey literature search was also completed (ERA) according to the CADTH Grey Matters checklist [35]. Google Scholar was used for searching for eligible papers that cited the included studies.

### Data extraction and synthesis

Data extraction was completed independently by EK, LJJS, LvB-V, BMH and ERA using a standardized summary table consisting of the following categories: author, title and year, country, design, population, clinical setting, outcome measures, low-value practice, intervention, targeted personnel or roles, control or comparator, use of low-value practice before or after intervention, and change in use of low-value practice. Data extraction was discussed in the research team for quality assurance purposes.

The findings from included studies were narratively synthesized. This synthesis was performed due to the variety of study designs among included studies and thus a meta-analysis was not feasible [36]. The narrative synthesis included familiarization, the development of a preliminary synthesis by organizing findings in tables. Then, relationships, patterns, and connections in the data were explored [36]. In addition, a subgroup analysis was done for interventions done in the USA separately.

## Results

### Search of the literature

**Fig. 1:**
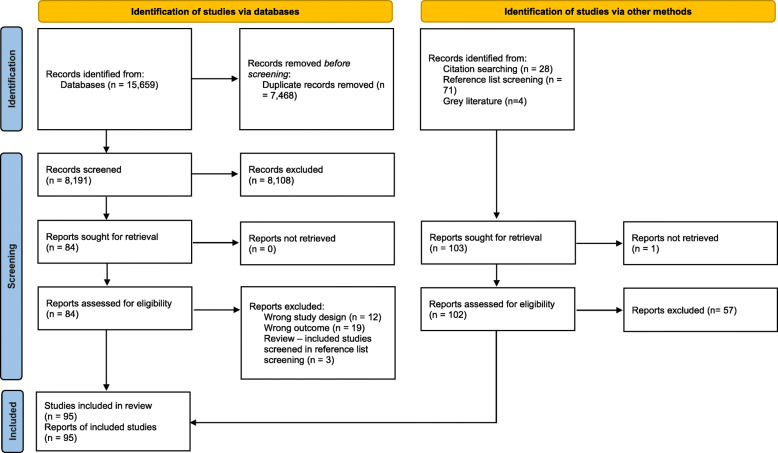
Flow diagram for record selection

As shown in Fig. 1, the electronic database searches resulted in 15,659 records. After the removal of 7468 duplicates, 8191 unique records were screened through title and abstract screening and 8108 records were excluded. An additional 103 records were identified through snowballing techniques and from the grey literature. A total of 186 articles were reviewed in full-text and 91 articles were excluded. Thus, 95 studies were included in the narrative synthesis. An overview of excluded studies with reason for exclusion is provided in Additional file 2.

### Quality of included studies

The 95 included studies [37–131] are summarized in Table 3. All included papers fulfill the screening questions in MMAT. Forty-six papers were given a full score in the MMAT appraisal. These are marked with a * in Table 3 (the full MMAT report is available in Additional file 3), while the others had one criterion unfulfilled, or lacked sufficient information in the report.

**Table 3:** Characteristics of the included studies, outcome of the intervention and quality assessment result

Author (year)	Country	Methods	Population	Clinical setting	Intervention	Low-value practice	Outcome of intervention(s)	Quality assessment
Arora et al. (2020) [126]	USA	Evaluation of a quality improvement project	1535 children	Emergency department	Education, decision support, and performance feedback	Head CT	**4.6%-points reduction in use**	*
Ashykian et al. (2019) [37]	USA	Retrospective review	1000 reports	Orthopedic and family practice departments	Education	Repeat radiographs for routine follow-up of osteoarthrosis	**50% reduction in use**	*
Bailey et al. (2013) [38]	USA	Longitudinal data analysis	800 patients	Emergency department	Health information exchange	Repeated lumbar or thoracic imaging	**64% lower odds of repeat**	*
Bailey, Wan et al. (2013) [39]	USA	Longitudinal data analysis	1252 patients	Emergency department	Health information exchange	Diagnostic neuroimaging	**62% lower odds of repeat**	*
Bairstow et al. (2010) [40]	Australia	Pre/post audit	1061 patients	Emergency department	Education, request forms	Chest CTA, abdominal XR, imaging with a provisional diagnosis of renal colic and XR despite negative Ottawa Ankle Rule	**13–28% reduction in use**	*
Baker et al. (2020) [41]	USA	Retrospective registry review	445 patients	Emergency department	Education and guideline	C-spine CT	**> 30% reduction in use**	*
Ballard et al. (2019) [127]	USA	Nonrandomized clinical trial - secondary analysis	3859 children	Emergency department	Electronic clinical decision support	Head CT	2.6% unsignificant reduction in use	*
Bhatia et al. (2013) [44]	USA	Prospective, time series analysis	1711 patients	Academic medical center	Education	Transthoracic echocardiography	**26% reduction in use**	
Bhatia et al. (2014) [42]	USA	Randomized Control Trial	88 internal medicine residents and 24 cardiology fellows	Academic medical center	Education	Transthoracic echocardiography	**62% reduction in use**	*
Bhatia et al. (2017) [43]	Canada/USA	Multicenter, randomized controlled trial	196 physicians	8 hospitals	Education and feedback	Transthoracic echocardiogram	**1.3% significant reduction in use**	*
Blackmore et al. (2011) [45]	USA	retrospective cohort study	6141 patients	Medical Center	Decision support system	Lumbar MRI, Brain MRI in headache, and sinus CT	**23.2–26.8% significant lower use**	
Bookman et al. (2017) [46]	USA	Longitudinal, before/after study	235,858 patient visits	5 emergency departments	Clinical decision support system	Head/c-spine CT, Chest CTA	*> 6% significant decrease in head and c-spine CT* *2% non-significant reduction chest CTA*	*
Boutis et al. (2013) [47]	Canada	Interrupted time series with pair matched control design.	2151 children	6 emergency departments	Education, reminders, and computerized decision support system	Ankle XR	**22% reduction in use**	*
Breakell et al. (2018) [48]	UK	Retrospective audit	101 children	District General Hospital	Education and guideline	Chest XR for Bronchiolitis	**16% reduction in use**	
Buntine et al. (2018) [49]	Australia	Before and after study	2931 scans	3 hospitals	Flowchart	Chest CTA and NM ventilation perfusion	**6 per 1000 scans reduction in use**	*
Carnevale et al. (2015) [128]	USA	Before-and-after study	29,395 encounters	Emergency department	Decision support and education	Several	**5–10% reduction in use**	*
Carpenter et al. (2020) [50]	USA	Retrospective chart review	1010 children	Hospital	Choosing Wisely	CT/MR or US in cryptorchidism	No significant difference	*
Chandra et al. (2019) [51]	Canada	Evaluation of a quality improvement project	37 physicians	Community teaching hospital	Didactic seminar, Survey	Imaging for low back pain	4%-points significant increase	*
Chang et al. (2018) [52]	USA	Before-and-after study	202 family physicians and 8 general internists	Clinics	Feedback reports	CT, MRI, and PET	**14.5% reduction in use**	*
Char et al. (2014) [53]	USA	Retrospective chart review	510 patients	Emergency department	Increased D-dimer threshold value and clinical probability assessment	Chest CTA	**7%-points Increase in diagnostic yield**	
Chen et al. (2020) [54]	USA	Before-and-after study	Physicians	Hospital	Alerts	Imaging for lower back pain	*9.6% reduction in total imaging rate and MRI (14.9%), No significant difference in use of CT/XR*	*
Chien et al. (2017) [55]	USA	Block randomized controlled trial	1205 clinicians	Multidisciplinary medical group	Financial incentives	CT or MRI for single headache or lower back pain, acute, uncomplicated rhinosinusitis, or DEXA for low risk for osteoporosis	No significant difference	*
Depinet et al. (2016) [56]	USA	Interrupted time series trial	1886 children	Urban tertiary care hospital	Clinical decision support system and pathway	Abdominopelvic CT/US	**2%-points increased use of US and 5%-points reduction in use of CT**	*
Doyle et al. (2019) [57]	USA	Randomized study	3524 practitioners	15 hospitals and 150 clinics	Best practice alerts	Several	**6% reduction in use**	
Drescher et al. (2011) [58]	USA	Before and after study	404 cases	Emergency department	Algorithm	Chest CTA	4.4% increase in use	*
Dudzinski et al. (2016) [59]	USA	Before and after study	65 cardiologists	Ambulatory cardiology practices in hospital	Education and feedback	Transthoracic echocardiography	**6%-points reduction in use**	*
Dunne et al. (2015) [60]	UK	Before and after study	5892 examinations	Hospital	Clinical decision support system	Chest CTA	**12.3% reduction in use**	*
Durand et al. (2013) [61]	USA	Randomized controlled trail	10 imaging tests	Tertiary teaching hospital	Cost display	Several	No significant difference	*
Ehrlichman et al. (2017) [129]	USA	Before-and-after study	104,454 patients	Emergency department	Feedback	Several	**2.3% reduction**	*
Fallon et al. (2016) [62]	USA	Prospective, longitudinal study	Children	Level I pediatric trauma center	Development of Trauma Protocol	Abdominal CT for abdominal trauma	**18%-points increase in diagnostic yield**	*
Ferguson et al. (2017) [63]	USA	Interventional improvement project	1 Emergency department	Hospital	Education and diagram	Abdominal XR constipation	**38%-points reduction**	*
Flamm et al. (2013) [64]	Austria	Non-randomized controlled trial with a historical control group	1363 patients	Hospital	PReOPerative evaluation’ (PROP)	Chest XR	**21.7%-points reduction**	
French et al. (2013) [65]	Australia	Cluster Randomized trail	112 general practitioners	Practices	Guideline/ facilitated interactive workshops	Lumbar CT or XR	No significant difference	
Gertz et al. (2016) [66]	USA	Before-and-after study	941 patients	Hospital	Computerized order entry tool	Cardiac stress tests with imaging	No significant difference	
Goldberg et al. (2011) [67]	USA	Retrospective cohort chart review	742 patients	Hospital	Guideline	Head CT	**16% reduction in use**	
Graves et al. (2018) [68]	USA	Interrupted time series	76,119 compensation claims	Regional	Policy implementation	Imaging for lower back pain	*5,6%-points significant decrease in use of MRI.* *2.46% increase in the use of XR* *No change in use of CT*	
Hardin et al. (2017) [69]	USA	Pre−/post-test design	339 patients	Hospital	Complex Care Map	CT scans	**62% reduction in use**	
Hess et al. (2018) [70]	USA	Cluster Randomized Trial	172 clinicians	Emergency department	Shared decision-making	Head CT (children)	No significant difference	
Hong et al. (2017) [71]	USA	Retrospective chart review	1,547,870 patients	Several	Choosing wisely	Imaging for lower back pain	**4% reduction in use**	
Hoo et al. (2011) [72]	USA	Retrospective chart review	457 examinations	Hospital	Mandatory clinical decision rule and selective d-dimer use	Chest CTA	**13%-points Increase in diagnostic yield**	*
Hui et al. (2014) [73]	USA	Prospective cohort study and retrospective review	762 patients	Hospital	Education and guideline	Pelvic US	**58% reduction in use**	
Hurley et al. (2017) [74]	USA	Before-and-after study	10,554 patients	Hospital	MUSIC imitative Collaborative	Bone scan and CT for prostate cancer	**4.5–7%-points reduction in use**	*
Ip et al. (2013) [78]	USA	Before-and-after study	1.8 million patient-months	Hospital	Computerized order entry tool with clinical decision support systems and accountability tools	Multiple	**12% reduction in use**	*
Ip et al. (2014) [75]	USA	Retrospective cohort study	21,445 LBP-related primary care visits	Academic quaternary care hospital	Clinical decision support system	Imaging for low back pain	**30.8% reduction in use**	*
Ip et al. (2015) [77]	USA	Before-and-after study	Emergency department patients	Hospital	Clinical decision support system	Head CT	**13.4% reduction in use**	*
Ip et al. (2017) [76]	USA	Before-and-after study	98,894 radiologyorders	Four institutions	Clinical decision support system	Several	**1%-point reduction in use**	
Jennings et al. (2017) [79]	USA	Evaluation of a quality improvement project	1346 Children	Community emergency department	Protocol, education, and individual feedback.	CT head of children, minor head injury	**12% reduction in use**	
Judkins et al. (2013) [80]	Australia	Retrospective chart review	659 children	Tertiary children’s hospital	NICE guidelines	Ultrasound urinary system, MUCG and dimercaptocsuccinic acid scintigraphy	**50% reduction in the use**	
Kandiah et al. (2020) [81]	Canada	Evaluation of a quality improvement project	4480 patients	Hospital	Education and information packages	MRI and CT of joints without red flags	**CT 43% reduction in use** **MRI 0.6% reduction of use**	*
Kanaan et al. (2013) [82]	USA	Retrospective chart review	200 patients	Tertiary emergency department	Education	Chest CTA	No significant difference	*
Keveson et al. (2017) [83]	USA	Evaluation of a quality improvement project	All invasive mechanical ventilator patients	Tertiary hospital	Education and change in routines/referral system	Daily CXR of ventilated patients	**64% reduction in use**	*
Kobes et al. (2020) [130]	Canada	Retrospective chart review	28 medical imaging sites	Mobile radiography	Guideline	Chest XR	**3.2% reduction in use**	*
Lacson et al. (2017) [84]	USA	Retrospective chart review	63,222 orders	Hospital	Clinical decision support system	Several	No significant difference	
Levitt et al. (2015) [120]	USA	Before-and-after study	415 patients	Hospital	Decision support and education	Stress echocardiography	**12%-points reduction in use**	*
Lu et al. (2012) [85]	USA	Retrospective chart review	267 patients	Hospital	Importing images from other institutions into PACS	Repeat imaging	**61%-points reduction in use**	
Luther et al. (2019) [86]	USA	Retrospective chart review	273 patients	Hospital	Standardized clinical assessment and management plans	Wrist XR	**60% reduction in use**	*
Masood et al. (2020) [87]	Canada	Evaluation of a quality improvement project	Parents in the emergency department	Tertiary care center	Education, guideline and checklist, patient handouts, and feedback	Head CT in adults	**7–14% reduction in use**	
McGrew et al. (2018) [88]	USA	Retrospective chart review	1934 children	Pediatric Level 2 Trauma Center	Guideline	Head and abdomen/pelvis CT pediatric trauma	**11.5–18.8% reduction in use**	
Mills et al. (2018) [121]	USA	Before-and-after study	7987 patients	Emergency department	Decision support	Chest CTA	**2.5% increased diagnostic yield**	*
Min et al. (2017) [89]	Canada	Retrospective chart review	4562 patients	Emergency department	Checklist	Imaging in low back pain	**22% reduction in use**	
Mittal et al. (2014) [91]	USA	Before and after design	3641 patients	Tertiary care hospital	Clinical practice guidelines	Chest XR	**14.6–20%-points reduction in use**	
Moriarity et al. (2015) [91]	USA	Retrospective chart review	33,311 patients	Hospital	Clinical decision support	Inpatient MR, CT, and NM	No significant difference	
Mulders et al. (2020) [92]	The Netherlands	Before-and-after comparative prospective cohort study	1261 patients	Emergency department	Amsterdam Wrist Rules	Wrist XR	**15%-points reduction in use**	
Mäenpää et al. (2011) [93]	Finland	Retrospective, longitudinal study	1 regional hospital	Hospital	Regional Health InformationExchange	Several	**16.4% reduction in use**	*
Nigrovic et al. (2015) [94]	USA	Multifaceted quality improvement initiative	Children < 21 years	Urban tertiary care academic center	Guideline development, feedback, and education	Head CT	**6%-points reduction in use**	*
O’Connor et al. (2014) [95]	USA	Prospective before and after study	28,420 CT orders	Tertiaryacademic medical center	Requiring a clinical justification to override a repeat CT alert	Several	**Prevented 1 in 13 scans**	*
Ong et al. (2013) [122]	USA	Before-and-after study	471 patients	Hospital	Algorithm and guideline	Chest CTA	**26% reduction in use**	*
Ostby et al. (2020) [96]	USA	Evaluation of a quality improvement project	235 patients	Emergency department	Specialist consolation before imaging	CT of gynecological cancer patients	**54%-points reduction in use**	*
Palen et al. (2019) [97]	USA	Stepped-wedge study	31,426 orders	several	Check boxes	Several	**Modest increase in appropriateness**	
Parikh et al. (2016) [98]	USA	Retrospective cohort study	220,539 patients	Hospital	Guideline	Chest XR	**6.4% significant reduction in use**	*
Poeran et al. (2019) [99]	USA	Retrospective interrupted time series	27,549 orders	Emergency department	Clinical decision support	Low appropriateness imaging	**9%-points reduction in use**	*
Prevedello et al. (2013) [100]	USA	retrospective cohort study	2891 patients	Emergency department	Alerts	Chest CTA	**2.2/1000 reduction in use**	*
Puffenbarger et al. (2019) [123]	USA	Retrospective chart review	556 visits	Emergency department	Education, guideline, and hand-outs	Head CT	**21.6%-points reduction in use**	*
Pugel et al. (2018) [101]	USA	Retrospective interrupted time series	213,532 consultations	Ambulatory care	Education, feedback and guidelines	DEXA and head CT	**23.4% reduction in use**	
Raja et al. (2012) [103]	USA	Retrospective cohort study	6838 patients	Quaternary care institution	Clinical decision support system	Chest CTA	**20.1% reduction in use**	*
Raja et al. (2015) [102]	USA	Prospective randomized controlled trial	2167 patients	Urban level 1 adult trauma center	Feedback	Chest CTA	**2/1000 reduction in use**	*
Reiter et al. (2018) [104]	Israel	Prospective cohort study	544 children	Pediatric Emergency department	Education, guideline cards at computers	Chest XR for Bronchiolitis	**20%-points reduction in use**	*
Rezaii et al. (2020) [105]	USA	Cohort study	27 practices, 4601 cases	Academic and private practices	Online educational material and feedback	Chest CTA, advanced imaging of low back pain, follow-up of adnexal cysts	**3% reduction in use**	*
Rosati et al. (2015) [106]	USA	Retrospective review	233 children	Level I trauma center	Guideline	C-spine CT	**23% reduction in use**	
Sclafani et al. (2010) [107]	USA	Retrospective chart review	1092 patients	Hospital	Education	Head CT, brain MRI and carotid US of syncope patients	No significant difference	
Shah et al. (2016) [108]	USA	Chart review	824 children	Emergency department	Diagnostic algorithm	Abdominal CT appendicitis	**51.2%-points reduction in use**	
Shelton et al. (2015) [131]	USA	Before-and-after study	2001 patients	Hospital	Feedback	Several	**38% reduction in use**	*
Singer et al. (2014) [109]	USA	Before and after trial design	34,961 children	Academic medical center	Opening a dedicated pediatric ED	Several	**3.2% reduction in use**	
Sodickson et al. (2011) [110]	USA	Retrospective chart review	1487 patients	Tertiary care, level I trauma center	Health InformationExchange	Several	**16–18% reduction in use**	
Sy et al. (2016) [111]	Canada	Evaluation of a quality improvement project	1492 patients	Intensive care unit	Education, posters, and change in order system	Chest XR	**26% reduction in use**	*
Tajmir et al. (2017) [124]	USA	Randomized controlled trail	613 patients	Hospital	Decision support	Ankle XR	Modest change in diagnostic yield	
Tyler et al. (2018) [112]	USA	Evaluation of a quality improvement project	2211 patients	Children’s hospital	Education, feedback, sign pledge, visualizing algorithm, guideline	Chest XR	**12.1%-points reduction in use**	
Vartanians et al. (2010) [113]	USA	Retrospective study	118,975 orders	Several	Change in ordering system	Several	**3.5%-points reduction in use**	*
Walen et al. (2016) [125]	USA	Prospective observational study	250 patients	Hospital	Wells-score documentation	Chest CTA	**6.6% increase in diagnostic yield**	
Walker et al. (2020) [114]	Canada	Retrospective chart review	302 Consultations	Primary care	Electronic communication system	Abdominal, musculoskeletal, neuro, and thoracic imaging	**28% reduction in use**	
Wang et al. (2018) [115]	USA	Retrospective chart review	3 clinics	Primary care	On-site and online education and feedback	Lumbar MRI	**3.7 MRIs reduced per month**	
Wu et al. (2020) [116]	USA	Evaluation of a quality improvement project	6441 Chest XRs	Medical Intensive care unit	Survey, journal club, discussions, posters, alerts in electronic referral system, education, pocket cards, electronic decision tool	Chest XR	**36.1% reduction in use**	
Xu et al. (2020) [117]	Canada	Retrospective review	400 referrals	Tertiary care center	Mandatory check list	Knee MRI	**48% reduction in use**	*
Zafar et al. (2019) [118]	USA	Randomized cohort study	54 providers	Tertiary academic health system	Algorithm and alerts	Low back imaging	No significant difference	*
Zamora-Flores et al. (2015) [119]	USA	Retrospective chart review	322 children	Rural community hospital	Guideline	Chest XR	No significant difference	

*CT* computed tomography, *CTA* *CT* angiography, *DEXA* dual-energy x-ray absorptiometry, *MMAT* Mixed Methods Appraisal Tool, *MRI* magnetic resonance imaging, *MUCG* micturating cystourethrogram, *NM* nuclear medicine, *PET* positron emission tomography, *US* ultrasound, *XR* X-ray

* Fulfill all MMAT criteria

*Italic = Mixed results*, Bold = Reduction in use/increase in rate of diagnostic yield, Normal = no significant difference/increase

### Characteristics of included studies

A majority of the included studies applied quantitative study designs. Retrospective chart reviews (*n* = 26) and uncontrolled before-after studies (*n* = 14) were the most common. Seventy-eight of the studies were conducted in the USA (82%). The setting of the studies included hospital (*n* = 40), emergency department (*n* = 24), or outpatient medical center (*n* = 18). Musculoskeletal (*n* = 26), neurological (*n* = 23) and vascular (*n* = 16) imaging were most commonly evaluated. The most targeted imaging examinations were chest CTA (*n* = 15) and head CT (*n* = 12). Further, pulmonary embolism (*n* = 15), lower back pain (*n* = 14), and minor head injury (*n* = 12) were the most commonly explored medical conditions.

### Interventions

**Fig. 2:**
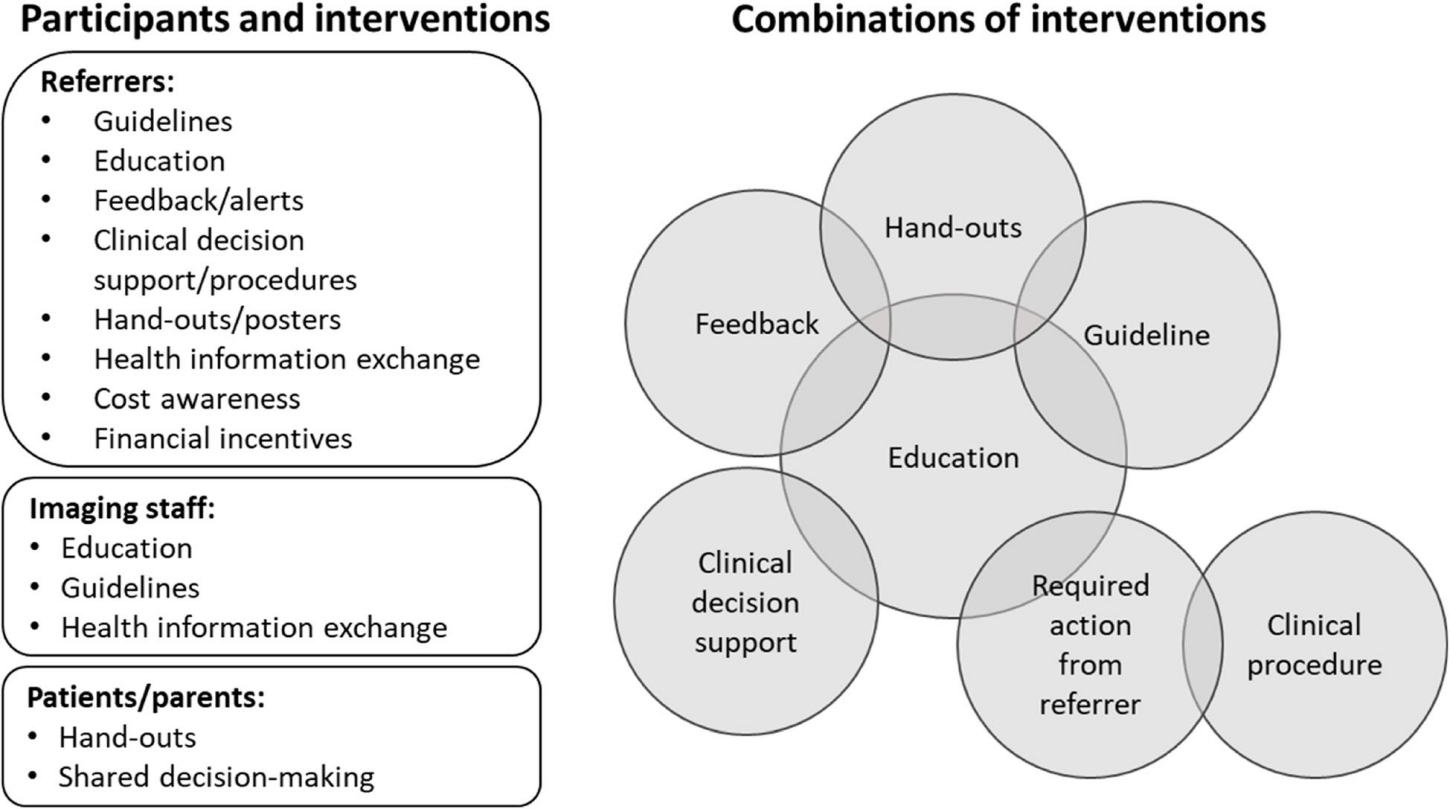
Overview of participants, interventions, and combinations in multi-component interventions in the included studies. Figure legend: To the left an overview of single interventions used for each participant group. To the right an illustration for how multi-component interventions were combined. Overlapping circles illustrate different combinations of two of more components

Guidelines (*n* = 28) and education (*n* = 28), either alone or in combination with other measures, were the most common interventions evaluated to reduce low-value imaging. The outcome measures reported in the included studies varied, with the number or rate of imaging examinations (*n* = 75) most frequently reported primary outcomes. A majority of studies (*n* = 61) used a single component intervention and most studies (*n* = 90) targeted referring physicians. An overview of participants exposed to the intervention (referring physicians, imaging staff, patients and/or family members), types of interventions, and combinations of components in multi-component interventions are presented in Fig. 2.

A variety of imaging modalities or patient diagnoses were targeted, and the primary outcomes varied among studies that reported improvements post-intervention. Among studies targeting several imaging modalities or diagnoses, 74–79% of the studies showed a reduction in use of low-value imaging. In contrast, studies targeting one specific modality only showed that targeting X-ray [37, 47, 48, 63, 64, 83, 86, 90, 92, 98, 104, 112, 119, 124, 130], CT [41, 46, 49, 53, 54, 58, 60, 62, 67, 69, 70, 77, 79, 82, 87, 88, 94–96, 101, 102, 106, 108, 121–123, 125–127, 129] or MRI [45, 68, 75, 105, 118] led to a 87, 86, and 83% reduction in low-value imaging, respectively. Few studies included other imaging modalities.

The most commonly targeted patient diagnosis was bronchiolitis [48, 90, 104, 112, 119], pulmonary embolism [49, 58, 60, 82, 100, 102, 121, 122, 125], and head injuries [67, 70, 77, 79, 87, 94, 101, 123, 126, 127]. In studies targeting these complaints, a reduction in use of low-value imaging were reported in 78–80% of the studies, while imaging in lower back pain [38, 45, 51, 53, 54, 65, 68, 71, 75, 89, 115, 118] were reduced in 58% of studies.

Among the 77 studies that reported improvements following the intervention [37–45, 47–49, 52, 53, 56, 57, 59, 60, 62–64, 67, 69, 71–81, 83, 85–90, 92–106, 108–117, 120–123, 125, 128–131], decreases in low-value imaging varied largely from < 1 to 62%. Of the remaining studies, three studies reported mixed results, where only some of the targeted low-value imaging examinations were reduced [46, 54, 68], and 16 studies showed a non-significant change or increase in the use of low-value imaging post-intervention [50, 51, 55, 58, 61, 65, 66, 70, 82, 84, 91, 107, 118, 119, 124, 127].

**Fig. 3:**
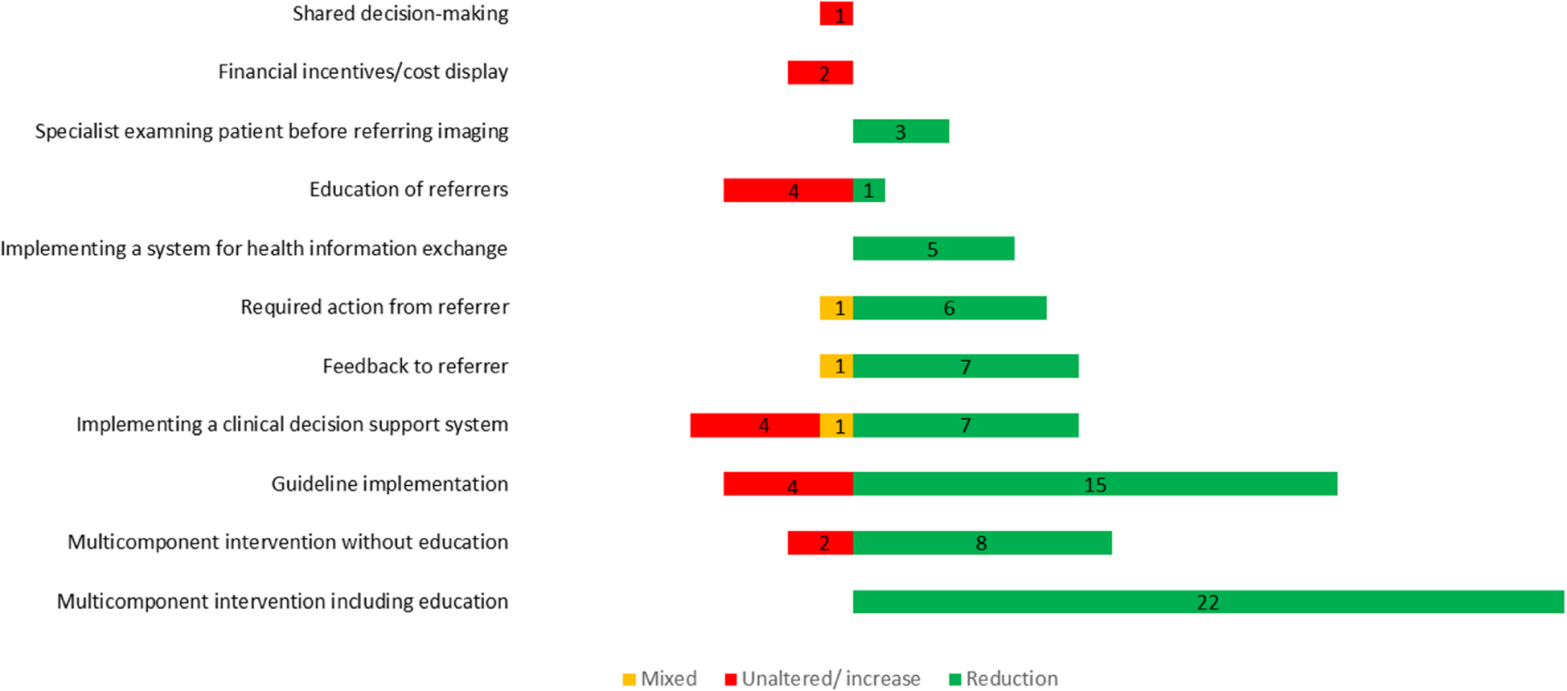
The number of studies and outcome of different types of interventions to reduce low-value imaging

Implementation of multi-component interventions (2 or more components in combination) reportedly reduced the use of low-value imaging among 94% of the included studies [40–44, 47, 48, 51, 53, 56, 59, 63, 72, 78, 79, 81, 83, 86, 87, 94, 101, 104, 105, 111, 115, 116, 118, 120, 122, 123, 126, 128]. Multi-component interventions were found to be more effective when education was one of the components. Following implementation of a single component intervention, 74% of included studies reported decreases in low-value imaging [37–39, 45, 46, 49, 50, 52, 54, 55, 57, 58, 60–62, 64–71, 74–77, 80, 82, 84, 85, 88–93, 95–100, 102, 103, 106–110, 113, 114, 117, 119, 121, 124, 125, 127, 129–131]. Data analyses based on the USA studies demonstrated similar results as 96% of multi-component and 68% of single-component interventions showed reduction in the use of low-value imaging. Thus, county of intervention does not affect the result alone. Implementation of guidelines or clinical decision support systems were the most effective single-component interventions [37–121, 126, 128, 130]. Furthermore, 23% of single-component interventions compared to 6% of multi-component interventions showed no statistically significant difference or an increase in the use of low-value imaging. In Fig. 3, the green bars represent studies with a reduction in the use of low-value imaging, red bars represent no significant change or increase, and orange bars represent mixed results.

Single-component interventions such as education, shared-decision-making, and financial measures alone often had no effect on use of low-value imaging [55, 61, 70, 82, 107]. Studies with more than 30% reduction in use of low-value imaging were both single-component (*n* = 11) and multi-component (*n* = 8) interventions [37–39, 41, 42, 44, 69, 73, 75, 80, 81, 83, 85, 86, 96, 108, 116, 117, 131]. All these studies targeted refereeing physicians while one also targeted imaging staff. Another targeted referrer, imaging staff, and patients. Of these studies, 16% were from countries other than the USA. Table 4 provides an overview of the type of interventions that resulted in more than a 30% reduction in low-value imaging.

**Table 4:** Overview of interventions with more than a 30% reduction in use of low-value imaging examinations

Components in interventions	Country	Setting	Reduction	Reference
Clinical decision support system	USA	Hospital	31%	[75]
Feedback to referrers	USA	Hospital	38%	[131]
Multiple measures for referrers, imaging staff, and patients	Canada	Hospital	43%	[81]
Required action from referrers	Canada	Tertiary care center	48%	[117]
Education	USA	Medical center	50%	[37]
Specialist involved in ordering examinations	USA	Hospital	54%-points	[96]
Education and guideline implementation for referrers and imaging staff	USA	Hospital/Emergency department	30–58%	[41, 73]
Combination of new clinical procedures	USA	Hospital	60%	[86]
Education, feedback, and hand-outs	USA	Academic medical center	30–62%	[42, 44]
Guideline implementation	USA, Australia	Hospital/tertiary hospital	50–62%	[69, 80, 108]
Education, alerts, and new procedure for referrers	USA	Hospital/tertiary hospital	35–64%	[83, 116]
Health information exchange	USA	Hospital	61–64% lower odds for repeat examination	[38, 39, 85]

## Discussion

A large body of literature evaluating the outcome of interventions aimed at reducing low-value imaging was identified through this systematic review. Broadly, most interventions were found to be effective, with multi-component interventions more frequently reported to be effective compared to single-component interventions. All studies evaluating multi-component interventions with an education component reported reductions in low-value imaging. Multi-component interventions targeting the participants on several points providing education and then feedback and reminders over a longer period seems to be effective as change takes knowledge, motivation, and time [132]. Single-component interventions, particularly guideline implementation, clinical decision support systems, feedback, or actions required from the referrers, showed reduction in use of low-value imaging in several studies but not in all. This might be caused by organizational differences, differences in the clinical setting, or participants motivation [132]. Shared decision-making, new referring procedures, and financial measures demonstrated no effect; however, these interventions were only evaluated in a limited number of studies. Targeting specific examinations for specific conditions (e.g., bronchiolitis), targeting referrers, and only targeting one imaging modality seemed to be more effective than targeting several modalities or examinations referred from a variety of referrer groups (e.g., lower back pain). There was also a variety of outcome measure used among included studies. The number or rate of low-value imaging was the most common. Others included appropriateness and diagnostic yield. This warrants caution when comparing outcomes between different types of interventions.

The present results are in line with previous systematic reviews on interventions to reduce low-value services in general [12, 26, 29–32] and with a previous scoping review on unnecessary imaging, diagnostic tests, and procedures in hospitals [28]. The results indicate great variation in outcomes for many interventions. This is in accordance with research on innovation and interventions suggesting that the formal and informal network in the organization, motivation, flexibility, and fitness to the internal culture and core values in the organization where interventions are implemented, were key factors for a successful and long-lasting change of clinical practice [132–135]. Whether or not an intervention is successful in reducing the use of low-value imaging would thus depend on a variety of factors. Comparing studies conducted in the USA to those from other countries showed no difference in type of interventions used or in the rate of studies demonstrating > 30% reduction in the use of low-value imaging. Thus, the effect of intervention seems to be dependent on local culture and health care organizations rather than the national health system alone. In addition, only a few interventions were directed against patients, which is somewhat surprising as patients are also identified as drivers in the use of low-value imaging [136, 137]. Further research should include the patient perspective and the role of the radiology department in interventions to reduce low-value imaging in addition to a review on cost-effectiveness of interventions to reduce low-value imaging. Further investigation should focus on how interventions can be adapted to the culture and core values of the providers of health services in different contexts.

Our study has several limitations. Publication bias may have been introduced as articles with negative or nonsignificant findings are less likely to be published. Among the included studies, few reported null or low effect. Most studies had an uncontrolled before-after design not considering that there may be a secular downtrend in the use of the low-value imaging examinations due to the attention in campaigns, such as Choosing Wisely. Thus, the outcome may be overestimated. Further, the review may be subject to contextual bias and have limited generalizability, as most of the studies were conducted in the USA. Accordingly, caution is warranted when inferring from and applying the results in different settings. The proportion of single-center studies and observational studies may enhance the overall positive effect of the interventions [26]. Yet another limitation is related to indirect outcome measures, as many publications focus on interventions’ impact on volume and not on value. This is understandable as the change of low-value utilization is a warranted measure, but one should notice that the value of these services is not assessed. Moreover, it may be argued that the spectrum of imaging that is targeted by the interventions is biased by the methods to assess intervention outcomes. Nonetheless, we report a wide variety of interventions targeting many examinations. There are reasons to believe that the interventions are targeted strategically. For example, interventions that are believed to be effective may be targeted towards examinations documented to be of low value.

It may also be argued that retrospective chart reviews are not proper intervention studies, but as they are used systematically to assess change in practice, we have included them in this review. Additionally, the included studies afford providers’ perspective and as indicated, few studies elicited patient preferences or included patient-reported outcome measures. In addition, the snowballing uncovered a few studies published in 2021 not included in our analysis due to the inclusion criteria. The results of these studies are in line with the studies included in the analysis and thus not including these did not reduce the strengths of the analysis in this review [138–142].

## Conclusions

This systematic review demonstrates that interventions to reduce low-value imaging can be very effective, but that there is a large variation in types of interventions and their outcomes. We found that multi-component interventions reported reduction in low-value imaging or increased diagnostic yield more frequently compared to single-component interventions. The context in which the intervention is introduced seems to be of vital importance for successful reduction of low-value imaging. Thus, in the future multi-component interventions that are adapted to the local context are more likely to be successful. Further research is needed to assess how interventions to reduce low-value imaging can best be adapted to specific contexts and how to reduce the use of low-value imaging cost-effectively.

## Supplementary Information

**Additional file 1.** Search strategy and hits from database searches.

**Additional file 2.** Table of excluded studies.

**Additional file 3.** MMAT registration forms.

## Data Availability

Not applicable.

## Abbreviations

CT: Computed tomography
CTA: Computed tomography angiography
DEXA: Dual-energy X-ray absorptiometry
MMAT: Mixed Methods Appraisal Tool
MRI: Magnetic resonance imaging
MUCG: Micturating cystourethrogram
NM: Nuclear medicine
PET: Positron emission tomography
US: Ultrasound
XR: X-ray
