# Supplementary material for: Interventions to reduce low-value imaging – a systematic review of interventions and outcomes
Source: BMC Health Serv Res. 2021 Sep 18;21:983. doi: 10.1186/s12913-021-07004-z (PMC8449221; doi:10.1186/s12913-021-07004-z)
Supplement: Supplementary file 1 — Additional file 1. Search strategy and hits from database searches. [file 12913_2021_7004_MOESM1_ESM.pdf]

Additional file 1

Medline 13.10.2020 – search strategy and hits

ch Journals Books Multimedia My Workspace Visible Body What's New

arch History (38)

# ▲	Searches	Results
1	diagnostic imaging/ or cardiac imaging techniques/ or imaging, three-dimensional/ or neuroimaging/ or radiography/ or radionuclide imaging/ or respiratory-gated imaging techniques/ or tomography/ or ultrasonography/ or whole body imaging/	700393
2	exp Radiology/	37142
3	(MRI or x-ray* or xray* or ultrasound* or mammography or ultrasonography or DEXA or DXA or CT or radiograph* or radiolog* or tomography or imaging).tw.	2287611
4	(CAT adj scan).tw.	873
5	(bone adj scan).tw.	6089
6	(Magnetic adj resonance adj imaging).tw.	244133
7	1 or 2 or 3 or 4 or 5 or 6	2666569
8	exp Health Services Misuse/ or exp Medical Overuse/	11668
9	(Unnecessar* or overuse* or Inappropriate* or wasted or low-value or overdiagn* or overutili* or misuse* or (Low adj value) or unwarrent or redundant).tw.	199161
10	(Choosing adj wisely).tw.	858

11	8 or 9 or 10	206790
12	7 and 11	28514
13	Animal/ not (animal/ and human/)	4709852
14	12 not 13	27980
15	limit 14 to ((danish or dutch or english or german or norwegian or swedish) and last 10 years)	16346
16	exp Health Planning/	350191
17	(reduc* or prevent* or stop* or replac* or abandon* or avoid* or deinvest* or de-invest or deadopt* or de-adopt* or deimplement* or de-implement* or restrict* or lower* or decrease* or (practice adj revers*) or educat* or guidel*).tw.	9173440
18	(academic adj detailing).tw.	568
19	16 or 17 or 18	9422593
20	15 and 19	10846
21	exp Mass Screening/	129223
22	(Unnecessary adj surger*).tw.	2162
23	(unnecessary adj biops*).tw.	1233
24	(mammography adj screening).tw.	3102
25	(lung adj cancer adj screening).tw.	2930
26	(unnecessary adj invasive adj procedure).tw.	20
27	(prenatal adj screening).tw.	2964
28	(case adj report).tw.	360519
29	(comment or editorial or letter).pt.	1919023
30	(Radioactive adj Waste).tw.	1519
31	(machine adj learning).tw.	34198
32	(deep adj learning).tw.	12132
33	(radio adj therapy).tw.	753
34	(optical adj imaging).tw.	8937
35	(soil or cell* or fetal or dentist* or denture*).tw.	5855720
36	(cancer adj screening).tw.	33027
37	21 or 22 or 23 or 24 or 25 or 26 or 27 or 28 or 29 or 30 or 31 or 32 or 33 or 34 or 35	8149890
38	20 not 37	7700

Embase 13.10.2020 - search strategy and hits

earch History (53)

# ▲	Searches	Results
1	exp diagnostic imaging/	197379
2	exp radiology/	50482
3	radiolo*.tw.	369890
4	radiograph*.tw.	270297
5	ultraso*.tw.	550793
6	MRI.tw.	428879
7	CT.tw.	589539
8	bone densitometry/	5885
9	exp mammography/	57885
10	exp X ray/	70161
11	exp x-ray computed tomography/	59778
12	exp nuclear magnetic resonance imaging/	987057
13	health services misuse.mp.	47
14	medical overuse.mp.	145
15	Unnecessar*.tw.	86663
16	overuse*.tw.	16885
17	Inappropriate*.tw.	98107
18	wasted.tw.	5446
19	low-value.tw.	4206
20	overdiagn*.tw.	5675
21	overutili*.tw.	1349
22	misuse*.tw.	27080
23	unwarrent.tw.	0
24	redundant.tw.	29061
25	"choosing wisely".tw.	1443
26	Animal/ not (animal/ and human/)	1084810
27	1 or 2 or 3 or 4 or 5 or 6 or 7 or 8 or 9 or 10 or 11 or 12	2548342
28	13 or 14 or 15 or 16 or 17 or 18 or 19 or 20 or 21 or 22 or 23 or 24 or 25	264836
29	27 and 28	34917

30	29 not 26	34807
31	limit 30 to (embase and (danish or dutch or english or german or norwegian or swedish) and (article or article in press or editorial or "review") and last 10 years)	10269
32	exp health care planning/	98727
33	(reduc* or prevent* or stop* or replac* or abandon* or avoid* or deinvest* or de-invest or deadopt* or de-adopt* or deimplement* or de-implement* or restrict* or lower* or decrease* or (practice adj revers*) or educat* or guidel*).tw.	11358658
34	(academic adj detailing).tw.	803
35	32 or 33 or 34	11427348
36	31 and 35	6855
37	exp mass screening/	247377
38	(Unnecessary adj surger*).tw.	2833
39	(unnecessary adj biops*).tw.	1810
40	(mammography adj screening).tw.	3710
41	(lung adj cancer adj screening).tw.	4466
42	(unnecessary adj invasive adj procedure).tw.	31
43	(prenatal adj screening).tw.	4068
44	(Radioactive adj Waste).tw.	2623
45	(machine adj learning).tw.	38053
46	(deep adj learning).tw.	13310
47	(radio adj therapy).tw.	1327
48	(optical adj imaging).tw.	10385
49	(soil or cell* or fetal or dentist* or denture*).tw.	6894897
50	(cancer adj screening).tw.	44271
51	(case adj report).tw.	426166
52	37 or 38 or 39 or 40 or 41 or 42 or 43 or 44 or 45 or 46 or 47 or 48 or 49 or 50 or 51	7522571
53	36 not 52	4688

Scopus 13.10.2020 - search strategy and hits

3,037 document results

((((TITLE-ABS-KEY ("Diagnostic imaging") OR TITLE-ABS-KEY (radiology) OR TITLE-ABS-KEY (mri) OR TITLE-ABS-KEY (x-ray OR xray) OR TITLE-ABS-KEY (ultrasound OR ultrasonography) OR TITLE-ABS-KEY (mammography) OR TITLE-ABS-KEY (dexa OR dxa) OR TITLE-ABS-KEY (ct) OR TITLE-ABS-KEY (radiograph*) OR TITLE-ABS-KEY (radiolog*) OR TITLE-ABS-KEY (tomography) OR TITLE-ABS-KEY (cat PRE/ scan) OR TITLE-ABS-KEY (bone PRE/ scan) OR TITLE-ABS-KEY ("Magnetic resonance imaging"))) AND ((TITLE-ABS-KEY ("Health services misuse") OR TITLE-ABS-KEY ("choosing wisely" OR unnecessar* OR overuse* OR inappropriate* OR wasted OR low-value OR overdiagn* OR overutili* OR misuse* OR unwarrent OR redundant)))) AND ((TITLE-ABS-KEY (reduc*) OR TITLE-ABS-KEY (prevent*) OR TITLE-ABS-KEY (stop*) OR TITLE-ABS-KEY (replace*) OR TITLE-ABS-KEY (abandon*) OR TITLE-ABS-KEY (de-invest*) OR TITLE-ABS-KEY (de-adopt*) OR TITLE-ABS-KEY (de-implement*) OR TITLE-ABS-KEY (restrict*) OR TITLE-ABS-KEY (lower) OR TITLE-ABS-KEY (decrease*)))) AND NOT ((TITLE-ABS-KEY ("Unnecessary surgery") OR TITLE-ABS-KEY ("Unnecessary biopsy") OR TITLE-ABS-KEY ("mammography screening") OR TITLE-ABS-KEY (soil) OR TITLE-ABS-KEY ("cancer screening") OR TITLE-ABS-KEY (fetal) OR TITLE-ABS-KEY ("case report") OR TITLE-ABS-KEY (cells) OR TITLE-ABS-KEY ("radioactive waste"))) AND (LIMIT-TO (SRCTYPE , "j")) AND (LIMIT-TO (DOCTYPE , "ar") OR LIMIT-TO (DOCTYPE , "re") OR LIMIT-TO (DOCTYPE , "ed")) AND (LIMIT-TO (SUBJAREA , "MEDI") OR LIMIT-TO (SUBJAREA , "HEAL") OR LIMIT-TO (SUBJAREA , "MULT") OR LIMIT-TO (SUBJAREA , "ECON") OR LIMIT-TO (SUBJAREA , "NURS") OR LIMIT-TO (SUBJAREA , "SOCI") OR LIMIT-TO (SUBJAREA , "DECI")) AND (LIMIT-TO (PUBYEAR , 2021) OR LIMIT-TO (PUBYEAR , 2020) OR LIMIT-TO (PUBYEAR , 2019) OR LIMIT-TO (PUBYEAR , 2018) OR LIMIT-TO (PUBYEAR , 2017) OR LIMIT-TO (PUBYEAR , 2016) OR LIMIT-TO (PUBYEAR , 2015) OR LIMIT-TO (PUBYEAR , 2014) OR LIMIT-TO (PUBYEAR , 2013) OR LIMIT-TO (PUBYEAR , 2012) OR LIMIT-TO (PUBYEAR , 2011) OR LIMIT-TO (PUBYEAR , 2010)) AND (LIMIT-TO (LANGUAGE , "English") OR LIMIT-TO (LANGUAGE , "German")) AND (EXCLUDE (EXACTKEYWORD , "Human Tissue") OR EXCLUDE (EXACTKEYWORD , "Histopathology") OR EXCLUDE (EXACTKEYWORD , "Unclassified Drug") OR EXCLUDE (EXACTKEYWORD , "Nonhuman") OR EXCLUDE (EXACTKEYWORD , "X Ray Diffraction") OR EXCLUDE (EXACTKEYWORD , "Blood") OR EXCLUDE (EXACTKEYWORD , "Scanning Electron Microscopy") OR EXCLUDE (EXACTKEYWORD , "Immunohistochemistry") OR EXCLUDE (EXACTKEYWORD , "Animals")) AND (EXCLUDE (LANGUAGE , "Spanish") OR EXCLUDE (LANGUAGE , "French") OR EXCLUDE (LANGUAGE , "Portuguese") OR EXCLUDE (LANGUAGE , "Italian") OR EXCLUDE (LANGUAGE , "Turkish") OR EXCLUDE (LANGUAGE , "Polish") OR EXCLUDE (LANGUAGE , "Croatian") OR EXCLUDE (LANGUAGE , "Chinese")) AND (EXCLUDE (LANGUAGE , "Bulgarian") OR EXCLUDE (LANGUAGE , "Russian") OR EXCLUDE (LANGUAGE , "Serbian") OR EXCLUDE (LANGUAGE , "Persian") OR EXCLUDE (LANGUAGE , "Ukrainian")) AND (EXCLUDE (EXACTKEYWORD , "Cancer Screening") OR EXCLUDE (EXACTKEYWORD , "Health Care Cost") OR EXCLUDE (EXACTKEYWORD , "Early Detection Of Cancer") OR EXCLUDE (EXACTKEYWORD , "Mass Screening") OR EXCLUDE (EXACTKEYWORD , "Antibiotic Agent") OR EXCLUDE (EXACTKEYWORD , "Pregnancy") OR EXCLUDE (EXACTKEYWORD , "Screening")) AND (EXCLUDE (SUBJAREA , "BIOC") OR EXCLUDE (SUBJAREA , "CENG") OR EXCLUDE (SUBJAREA , "DENT") OR EXCLUDE (SUBJAREA , "EART")) AND (EXCLUDE (SUBJAREA , "CHEM") OR EXCLUDE (SUBJAREA , "ECON") OR EXCLUDE (SUBJAREA , "ENER") OR EXCLUDE (SUBJAREA , "ENVI")) AND (EXCLUDE (SUBJAREA , "IMMU") OR EXCLUDE (SUBJAREA , "MATE") OR EXCLUDE (SUBJAREA , "MATH") OR EXCLUDE (SUBJAREA , "PHYS") OR EXCLUDE (SUBJAREA , "SOCI") OR EXCLUDE (SUBJAREA , "PSYC") OR EXCLUDE (SUBJAREA , "PHAR") OR EXCLUDE (SUBJAREA , "AGRI"))

View less

Edit Save Set alert Set feed

Cochrane 13.10.20 - search strategy and hits

earch/search-manager

#1	MeSH descriptor: [Radiology] explode all trees	MeSH	197
#2	MeSH descriptor: [Diagnostic Imaging] explode all trees	MeSH	47176
#3	MRI	Limits	23894
#4	x-ray*	Limits	19425
#5	ultrasound*	Limits	31048
#6	mammography	Limits	2139
#7	ultrasonography	Limits	15530
#8	DEXA or DXA	Limits	4389
#9	CT	Limits	276446
#10	radiograph*	Limits	23500
#11	radiolog*	Limits	23797
#12	tomography	Limits	27137
#13	imaging	Limits	69343
#14	{OR #1-#13}	Limits	400054
#15	MeSH descriptor: [Health Services Misuse] explode all trees	MeSH	238
#16	MeSH descriptor: [Medical Overuse] explode all trees	MeSH	168
#17	Unnecessar*	Limits	4489
#18	overuse*	Limits	1197
#19	Inappropriate*	Limits	6649
#20	wasted	Limits	336
#21	"low-value"	Limits	141
#22	overdiagn*	Limits	441
#23	overutili*	Limits	58
#24	misuse*	Limits	2128

#25	unwarrent	Limits	0
#26	redundant	Limits	444
#27	"Choosing wisely"	Limits	55
#28	{OR #15-#27}<br>with Cochrane Library publication date from Jan 2010 to Sep 2020, in Cochrane Reviews	Limits	2619
#29	#14 AND #28<br>with Cochrane Library publication date from Jan 2010 to Sep 2020, in Cochrane Reviews	Limits	1214
#30	Reduc*	Limits	409981
#31	Avoid*	Limits	30107
#32	Prevent*	Limits	239674
#33	Stop*	Limits	26713
#34	replace*	Limits	38825
#35	abandon*	Limits	2812
#36	De-invest*	Limits	3664
#37	De-adopt*	Limits	37
#38	De-implement*	Limits	122
#39	restrict*	Limits	35632
#40	lower*	Limits	231801
#41	decrease*	Limits	218286
#42	educate*	Limits	3580
#43	guidel*	Limits	44536
#44	{OR #30-#43}	Limits	808105
#45	#29 AND #44	Limits	1214
#46	MeSH descriptor: [Mass Screening] explode all trees	MeSH	3759
#47	#45 NOT #46	Limits	1206
#48	MeSH descriptor: [Environmental Pollution] explode all trees	MeSH	3439
#49	#47 NOT 48	Limits	234
#50	Type a search term or use the S or MeSH buttons to compose	S MeSH Limits	N/A

Clear all

Highlight orphan lines
