# Supplementary material for: Interventions to reduce low-value imaging – a systematic review of interventions and outcomes
Source: BMC Health Serv Res. 2021 Sep 18;21:983. doi: 10.1186/s12913-021-07004-z (PMC8449221; doi:10.1186/s12913-021-07004-z)
Supplement: Supplementary file 2 — Additional file 2. Table of excluded studies. [file 12913_2021_7004_MOESM2_ESM.pdf]

Additional file 2: Table of excluded papers

| Author | Year | Title | Reason for exclusion |
|---|---|---|---|
| Ranta A, et al. | 2014 | Utility of a primary care based transient ischemic attack electronic decision support tool: a prospective sequential comparison | Wrong outcome |
| Yousem DM. | 2012 | Combating overutilization. Radiology Benefits Managers Versus Order Entry Decision Support | Wrong study design |
| Langer JM, et al. | 2015 | Reducing Unnecessary Portable Pelvic Radiographs in Trauma Patients: A Resident-Driven Quality Improvement Initiative | Wrong study design |
| Ingraham B, et al. | 2016 | Reduction in High-End Imaging Utilization With Radiology Review and Consultation | Wrong study design |
| Anandalwar SP, et al. | 2016 | Eliminating unnecessary routine head CT scanning in neurologically intact mild traumatic brain injury patients: implementation and evaluation of a new protocol | Wrong outcome |
| Richards SE, et al. | 2014 | A Clinical Process Change and Educational Intervention to Reduce the Use of Unnecessary Preoperative Tests | Wrong outcome |
| Schiavoni KH, et al. | 2016 | How Primary Care Physicians Integrate Price Information into Clinical Decision-Making | Wrong outcome |
| Jenkins HJ, et al. | 2018 | Using behavior change theory and preliminary testing to develop an implementation intervention to reduce imaging for low back pain | Wrong study design |
| Daum P. | 2013 | Improving the investigation of suspected deep vein thrombosis in the Emergency Department | Wrong study design |
| Ioos et al. | 2011 | An integrated approach for prescribing fewer chest x-rays in the ICU | Wrong study design |
| Hankin RA, Jones SP | 2020 | The impact of educational interventions on clinicians' knowledge of radiation protection: An integrative review | Wrong outcome |
| Riggs KR, et al. | 2017 | Can Appealing to Patient Altruism Reduce Overuse of Health Care Services? An Experimental Survey | Wrong study design |
| Vest JR, et al. | 2015 | Image Sharing Technologies and Reduction of Imaging Utilization: A Systematic Review and Meta-analysis | Studies screened for inclusion |
| Muhiy Addin R, et al. | 2020 | The Impact of Clinical Decision Support Systems (CDSS) on Physicians: A Scoping Review | Studies screened for inclusion |
| Deakyne SJ, et al. | 2015 | Development, Evaluation and Implementation of Chief Complaint Groupings to Activate Data Collection | Wrong outcome |
| Fenton JJ, et al. | 2016 | Promoting Patient-Centered Counseling to Reduce Use of Low-Value Diagnostic Tests | Wrong outcome |

| | | | |
|---|---|---|---|
| | | A Randomized Clinical Trial | |
| Gruber J, et al. | 2020 | The effect of increased cost-sharing on low-value service use | Wrong study design |
| Jarvik JG, et al. | 2020 | The Effect of Including Benchmark Prevalence Data of Common Imaging Findings in Spine Image Reports on Health Care Utilization Among Adults Undergoing Spine Imaging. A Stepped-Wedge Randomized Clinical Trial | Wrong outcome |
| Kullgren JT, et al. | 2018 | Precommitting to choose wisely about low-value services: a stepped wedge cluster randomised trial | Wrong outcome |
| Camissa C, et al. | 2011 | Engaging Physicians in Change: Results of a Safety Net Quality Improvement Program to Reduce Overuse | Wrong outcome |
| Arditi C, et al. | 2017 | Computer-generated reminders delivered on paper to healthcare professionals: effects on professional practice and healthcare outcomes | Wrong outcome |
| Bai L, et al. | 2020 | A systematic literature review on unnecessary diagnostic testing: The role of ICT use | Wrong outcome |
| Boutis K, et al. | 2015 | Cost Consequence Analysis of Implementing the Low Risk Ankle Rule in Emergency Departments | Wrong study design |
| Bhatia RS, et al. | 2014 | Educational Intervention to Reduce Inappropriate Transthoracic Echocardiograms: The Need for Sustained Intervention | Wrong study design |
| Reyes M, et al. | 2017 | Choosing Wisely Campaign: Report Card and Achievable Benchmarks of Care for Children's Hospitals | Wrong study design |
| Suman A, et al. | 2020 | A systematic review of the effectiveness of mass media campaigns for the management of low back pain | Studies screened for inclusion |
| Psoter KJ, et al. | 2014 | Effect of an Image-Sharing Network on CT Utilization for Transferred Trauma Patients: A 5-Year Experience at a Level I Trauma Center | Wrong outcome |
| Ross SE, et al. | 2013 | Effects of health information exchange adoption on ambulatory testing rates | Wrong outcome |
| Vest JR, et al. | 2014 | Health Information Exchange and the Frequency of Repeat Medical Imaging | Wrong outcome |
| Heubner L, et al. | 2019 | Using Digital Health to Support Best Practices: Impact of MRI Ordering Guidelines Embedded Within an Electronic Referral Solution | Wrong study design |
| Fleuren M, et al. | 2010 | Implementation of a shared care guideline for back pain: effect on unnecessary referrals | Wrong outcome |

| | | | |
|---|---|---|---|
| Chien L, et al. | 2019 | Reducing low value services in surgical inpatients in Taiwan: Does diagnosis-related group payment work? | Wrong outcome |
| Gupta A, et al. | 2014 | Effect of clinical decision support on documented guideline adherence for head CT in emergency department patients with mild traumatic brain injury | Wrong outcome |
| Kost A, et al. | 2015 | Clinical Decisions Made in Primary Care Clinics Before and After Choosing Wisely | Wrong outcome |
